# Bio-Performance of Hydrothermally and Plasma-Treated Titanium: The New Generation of Vascular Stents

**DOI:** 10.3390/ijms222111858

**Published:** 2021-11-01

**Authors:** Metka Benčina, Niharika Rawat, Katja Lakota, Snežna Sodin-Šemrl, Aleš Iglič, Ita Junkar

**Affiliations:** 1 Department of Surface Engineering, Jožef Stefan Institute, Jamova 39, SI-1000 Ljubljana, Slovenia; metka.bencina@ijs.si; 2 Laboratory of Physics, Faculty of Electrical Engineering, University of Ljubljana, Tržaška 25, SI-1000 Ljubljana, Slovenia; niharika.rawat@fe.uni-lj.si (N.R.); ales.iglic@fe.uni-lj.si (A.I.); 3 Department of Rheumatology, University Medical Centre Ljubljana, Vodnikova 62, SI-1000 Ljubljana, Slovenia; katja.lakota@guest.arnes.si (K.L.); ssodin1@yahoo.com (S.S.-Š.); 4 Laboratory of Clinical Biophysics, Department of Orthopaedics, Faculty of Medicine, University of Ljubljana, Zaloška 9, SI-1000 Ljubljana, Slovenia

**Keywords:** cardiovascular disease, metallic stents, hydrothermal treatment, non-thermal plasma treatment, TiO_2_

## Abstract

The research presented herein follows an urgent global need for the development of novel surface engineering techniques that would allow the fabrication of next-generation cardiovascular stents, which would drastically reduce cardiovascular diseases (CVD). The combination of hydrothermal treatment (HT) and treatment with highly reactive oxygen plasma (P) allowed for the formation of an oxygen-rich nanostructured surface. The morphology, surface roughness, chemical composition and wettability of the newly prepared oxide layer on the Ti substrate were characterized by scanning electron microscopy (SEM) with energy-dispersive X-ray analysis (EDX), atomic force microscopy (AFM), X-ray photoelectron spectroscopy (XPS) and water contact angle (WCA) analysis. The alteration of surface characteristics influenced the material’s bio-performance; platelet aggregation and activation was reduced on surfaces treated by hydrothermal treatment, as well as after plasma treatment. Moreover, it was shown that surfaces treated by both treatment procedures (HT and P) promoted the adhesion and proliferation of vascular endothelial cells, while at the same time inhibiting the adhesion and proliferation of vascular smooth muscle cells. The combination of both techniques presents a novel approach for the fabrication of vascular implants, with superior characteristics.

## 1. Introduction

Cardiovascular diseases (CVD) are currently the leading cause of death worldwide [1,2] and account for 45% of all deaths in Europe (4 million people) [3]. In 2016, approximately 17.6 million deaths were attributed to CVD globally, but by 2030, it is predicted that 23.6 million people will die from CVD annually [4]. Presumably, more than 60 million people in the EU live with CVD, and almost 13 million cases are diagnosed per year [5]. SARS-CoV-2 infection even worsens the cardiovascular disease burden, since it can lead to heart complications, such as arrhythmia, cardiac injury, heart failure and pulmonary embolism [5].

For patients affected by CVD, the implantation of a drug-eluting stent (DES) is currently the most commonly accepted treatment [6]. DESs have demonstrated superiority to bare-metal stents (BMSs) concerning reduced neointimal hyperplasia (restenosis) [7], however, delayed endothelization and an increased risk of late stent thrombosis was detected with the first generation of these devices [8,9]. The durable polymeric coatings applied in DES that served as a platform for hosting the drug of choice actually contributed to tissue acidification and inflammation, which led to late stent thrombosis [10] and the need for dual antiplatelet therapy. The new generation of stents coated with biodegradable [9] polymer were developed to overcome these issues, especially the persistent inflammation of the vessel wall caused by the continued presence of drugs [9]. Although studies showed promising results, late stent thrombosis and dual antiplatelet therapy could not be avoided [11]. More recently, bioresorbable vascular stents or scaffolds (BRS) were developed and are already available on the market. These types of scaffolds are not permanent, and their main advantage is that they offer temporary mechanical support in the vessel to prevent immediate restenosis and vascular recoil, while the long-term risk of complications due to the metallic stent matrix is eliminated. However, their superiority over DES is not yet clear, as long-term studies have not been conducted [9,12,13].

Blood-contacting medical devices made of metal, such as vascular implants, therefore still lack the desired biocompatibility. Despite intensive research and considerable advances in the surface modifications of metals, the implantation of metallic blood connecting devices still presents a risk of surface-induced thrombosis and, in the case of vascular stents, also restenosis. The main clinical risk after stent insertion, therefore, remains high platelet adhesion and activation, directly connected to stent-thrombosis; at the site of injury, which is common in stenting procedures, platelets quickly adhere and start to form a plug and blood coagulation that leads to vessel occlusion [14]. Besides, the uncontrolled proliferation of smooth muscle cells with high risk leads to the narrowing of a blood vessel and restricts blood flow [15]. These conditions can further lead to heart attack and stroke [16,17]. The development of stent surfaces strives to reach the biocompatibility level that would overcome serious clinical problems. Stent/implant surfaces of new generations should firstly and foremost inhibit the adhesion and activation of platelets and inhibit the proliferation and migration of smooth muscle cells, while promoting the viability and integrity of the endothelial cell layer.

Various surface modification approaches have been proposed to enhance the biocompatibility of stents, mainly based on multiple types of coatings, for instance organic (polymeric) or inorganic (ceramic layers such as titanium dioxide) [11,18,19,20,21,22,23,24]. Such coatings can alter the physicochemical characteristics of the surface, such as morphology, roughness, wettability and surface chemistry, which can affect the interactions with biological material. The nanostructuring of metallic surfaces can be achieved by various techniques, for instance electrochemical anodization [18,19], non-thermal plasma treatment [25], electrospinning [26,27], sandblasting [28,29] and hydrothermal treatment [30,31]. The fabrication of nano-sized metallic surfaces significantly influences cellular adhesion, proliferation and differentiation [19,32], as well as the adhesion and activation of platelets [33,34]. Contradictory results can be found in the literature regarding the influence of surface wettability and adhesion of cells; probably the main reason for this is that other surface features (surface roughness, surface chemistry) significantly contribute to biological response [35,36].

The present study aimed to investigate the effect of altered surface properties of the Ti substrate on interactions with blood platelets and vascular cells. The nanostructurization, combined with non-thermal plasma treatment, enabled the formation of a surface with superior morphological and chemical properties, especially appropriate for use as vascular stents. The titanium surface has been modified through a combination of hydrothermal treatment and treatment with reactive oxygen species (non-thermal oxygen plasma). Such a technique provides the formation of a nanostructured titanium oxide layer with enhanced oxygen concentration on the surface of a metallic substrate. Moreover, treatment with reactive oxygen plasma enables superhydrophilicity, which also influences the biological response. The efficacy of the hydrothermal and plasma treatment of Ti substrates has been established through hemocompatibility studies and the evaluation of human coronary artery endothelial cell (EC) and human coronary artery smooth muscle cell (SMC) proliferation.

## 2. Results

The results of WCA analysis of the Ti foil (Ti), plasma-treated Ti foil (Ti + P), hydrothermally treated Ti foil (Ti HT) and hydrothermally and plasma-treated Ti foil (Ti HT + P) are presented in Table 1. All plasma-treated surfaces were fully hydrophilic, as the water fully covered the surface and the contact angle was below the detection of our method (<5°). Similar goes for the freshly prepared HT samples; these surfaces were also fully wettable (Table 1).

**Figure 1:**
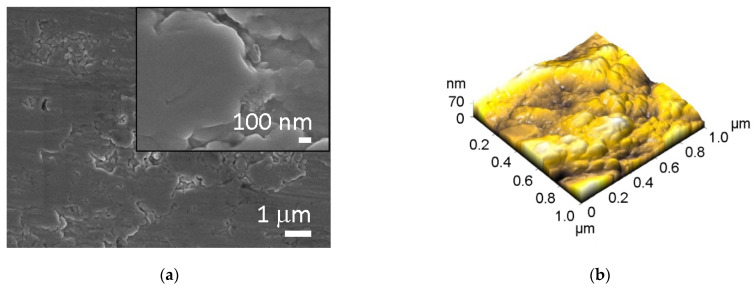

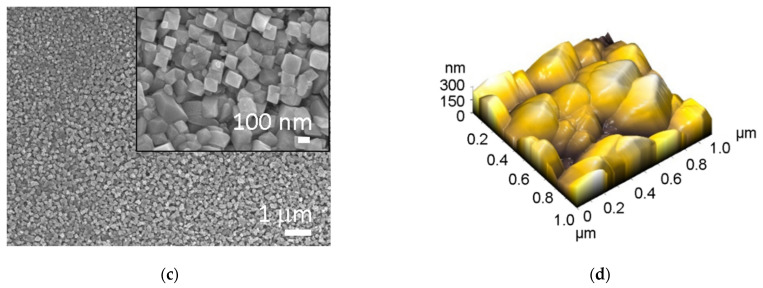
SEM and AFM images of: (**a**,**b**) untreated Ti foil (Ti), and (**c**,**d**) hydrothermally treated Ti (Ti HT), respectively.

Concerning the morphology, all surfaces were analyzed by SEM and AFM. The micrographs of untreated Ti and Ti HT are presented in Figure 1, while data for plasma-treated surfaces are not presented, as no changes in morphology were observed after plasma treatment. The untreated Ti surface has no defined surface topography; some microgrooves can be present on the surface probably due to the manufacturing process (Figure 1a), while the morphology of the hydrothermally treated surface (Ti HT) is nanostructured (composed of octahedral particles with a size of 30–200 nm) as can be seen in Figure 1c.

Atomic force microscopy (AFM) analysis was conducted to obtain detailed information about the surface roughness of the samples. The analysis of the pristine titanium surface (Ti) and the hydrothermally (Ti HT) treated surface is presented in Figure 1b,d. The analysis of Ti + P and Ti HT + P is not shown, as no morphological changes after plasma treatment were detected on these surfaces. The 3D image of surface topography (Figure 1b) reveals that plain Ti foil has no defined topography; the estimated surface roughness (Ra) is about 11.7 nm for the 1 × 1 µm^2^ scan area. On the other hand, much higher roughness (49 nm) was measured for the hydrothermally treated surface (Ti HT) for the same scan area. In the case of hydrothermal treatment, the evaluated height of the octahedral particles is between 180 and 300 nm. Thus, the main difference between Ti and Ti HT surface is that the TiHT exhibits homogenous structured nanotopography, which may significantly influence biological response.

As determined from X-ray photoelectron (XPS) analysis (Table 2), the untreated Ti substrate consists of 48.5 at.% of oxygen, 19.5 at.% of titanium and 32.0 at.% of carbon. Oxygen concentration on the surface of Ti + P and Ti HT + P increased in comparison to pristine Ti and Ti HT; plasma-treated surfaces contained 68.5 at.% and 61.1 at.% of oxygen, respectively. This further indicates a higher concentration of titanium oxide on these surfaces. The carbon concentration on the sample’s surfaces decreases in the following order Ti > Ti HT > Ti HT + P > Ti + P, which confirms that plasma significantly reduces carbon contamination. The pristine Ti foil has the highest carbon contamination (32.0 at.%), followed by the hydrothermally treated surface (about 31 at %), while the lowest carbon concentration was detected on plasma-treated surfaces (~16 at.%). Plasma treatment significantly reduced carbon on the surface, while no significant difference in the concentration of Ti was detected between the Ti HT and Ti HT + P surfaces (20.0 at.% and 22.3 at.%, respectively). By comparing the C/O and Ti/O ratios, it can be observed that the C/O ratio is the lowest for Ti HT + P, while the highest Ti/O ratio was observed for Ti HT (Table 2).

The elemental composition on the surfaces was examined also by EDX, in which the depth of analysis is larger (~1–2 μm) than for XPS technique (~5 nm). By EDX analysis, carbon was not detected on the samples, which confirms that carbon is present only in the top surface layer. The concentration of titanium and oxygen increased compared to XPS analysis for hydrothermally treated samples: Ti HT (Ti = 43.4 at.%, O = 56.5 at.%) and Ti HT + P (Ti = 37.5 at.%, O = 62.5 at.%). On the surface of Ti HT, a small amount of potassium (K = 0.1 at.%), used in the hydrothermal synthesis, was detected by EDX analysis. Only Ti (100% at.%) was detected on the untreated Ti foil and plasma-treated Ti (Ti + P).

**Figure 2:**
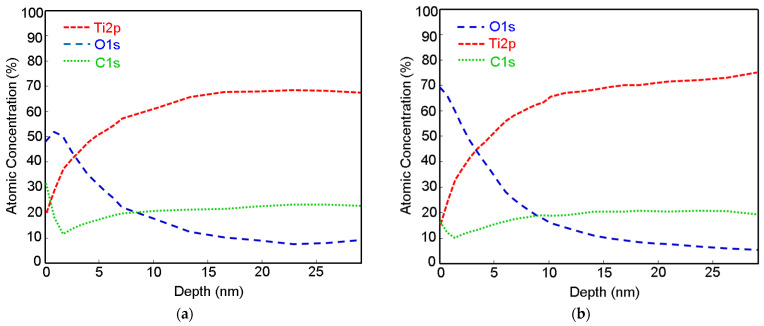

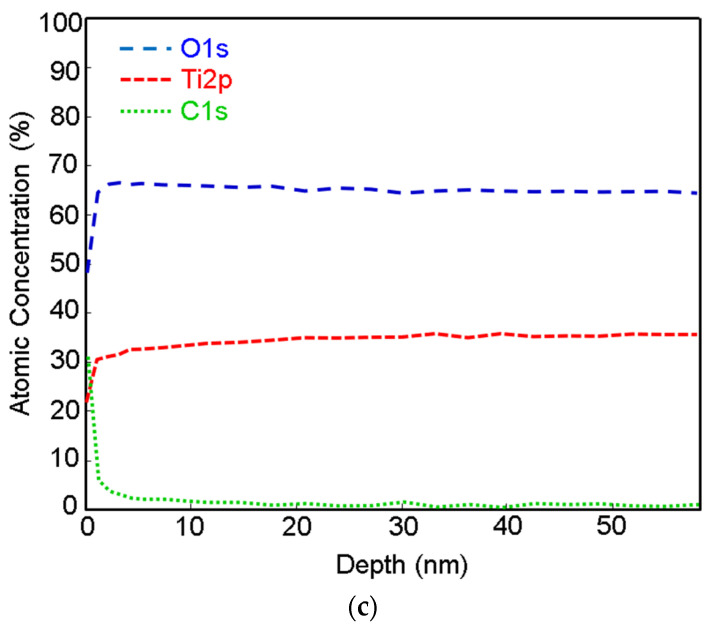
XPS depth profile analysis of the (**a**) untreated Ti substrate (Ti), (**b**) plasma-treated Ti substrate (Ti + P) and (**c**) hydrothermally treated Ti substrate (Ti HT).

The depth profiles of Ti, Ti + P and Ti HT obtained by XPS are presented in Figure 2 (data for Ti HT + P not shown, as no significant difference compared to the HT sample were detected). From the profile analysis, it can be observed that the oxide layer is present on the top surface. The naturally formed titanium oxide layer can be detected on the Ti foil (Figure 2a), with a thickness of about 3 nm. After treatment of the Ti foil with plasma, no significant increase in the thickness of the titanium oxide layer was observed, as seen in Figure 2b; however, the initial concentration of oxygen on the plasma-treated surface (Ti + P) increased for ~20 at.% compared to untreated Ti. The oxide layer on the Ti + P (~3.5 nm) is slightly thicker than on Ti (~3 nm). On all samples (Ti, Ti + P and Ti HT), the carbon appears at the top surface, as evident from Figure 2a–c, presumably due to contamination from air. On Ti HT, practically no decrease in oxygen was observed, even at a depth of 55 nm, which indicates the formation of a thick titanium oxide layer after hydrothermal treatment, which remains the same even after plasma treatment. Given that the ratio of Ti/O, determined from XPS analysis, is 0.41 (Table 2) and the shape of high-resolution Ti 2p peak (not shown), the Ti HT consists of a TiO_2_ layer.

The physicochemical properties of titanium surfaces include wettability, influence protein binding, cell adhesion and proliferation [37,38]. Therefore, wettability studies (WCA) were performed on the samples before the in vitro studies. It can be seen from Table 1 that the surface of Ti is hydrophobic (WCA = 97.6°). Contrarily, the contact angle for Ti HT and Ti HT + P measured 2 h after the synthesis (in the same time period the biological tests were performed) is below 5°, which is typical of a superhydrophilic surface.

**Figure 3:**
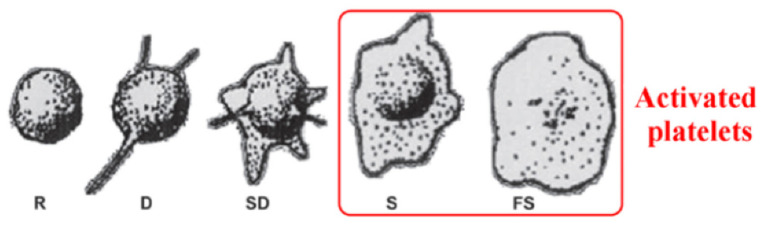
Copyright 2014 American Chemical Society.

To study hemocompatibility, the samples were incubated with whole blood, and afterward, the number and morphology of platelets were examined on the surface of Ti, Ti + P, Ti HT and Ti HT + P. Platelet adhesion and activation can be determined by counting the number of attached cells, as well as by observing the morphological changes. The stages of platelet shape from the least activated to fully activated platelets can be distinguished as round (R), dendritic (D), spread dendritic (SD), spread (S) and fully spread (FS) (Figure 3), in which S and FS are considered the activated form of platelets (Figure 3) [39].

**Figure 4:**
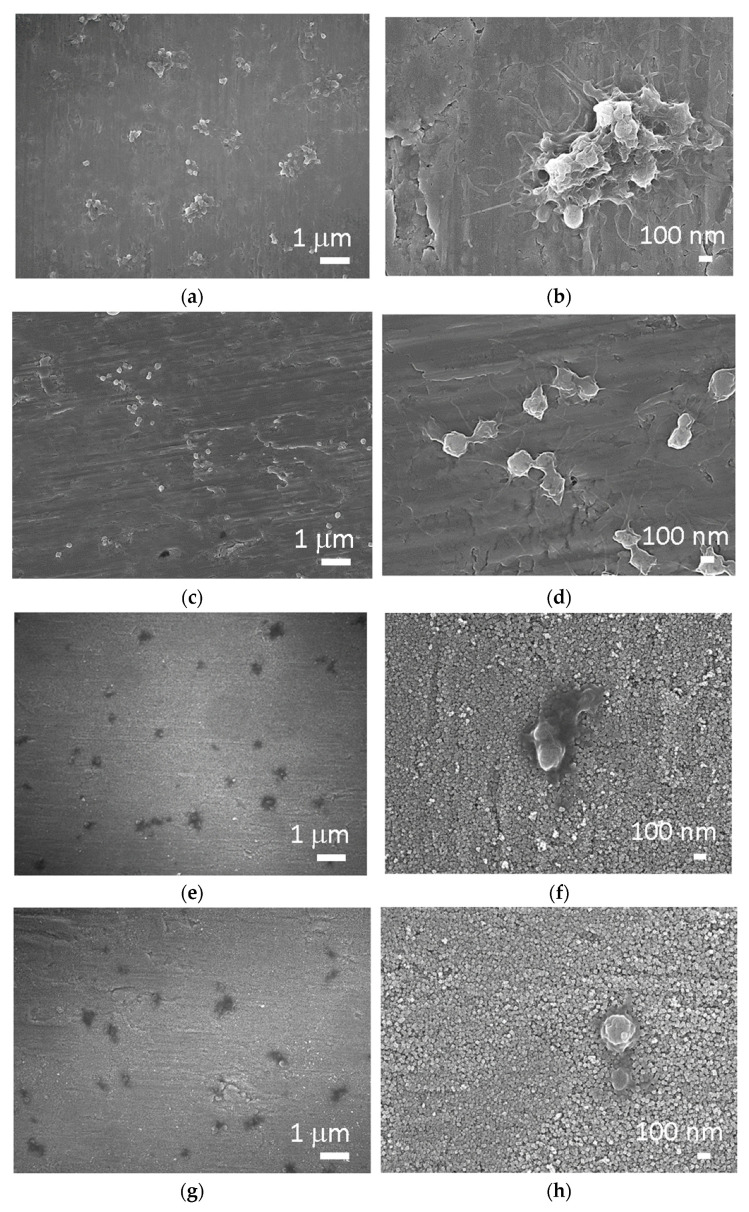
SEM images of (**a**,**b**) Ti foil, (**c**,**d**) Ti foil treated with plasma (Ti + P), (**e**,**f**) hydrothermally treated Ti foil (Ti HT) and (**g**,**h**) hydrothermally/plasma-treated Ti foil (Ti HT + P) after incubation with whole blood.

The interaction of materials and platelets analyzed by SEM is presented in Figure 4. It can be observed in Figure 4a,b that platelets attach to the surface of untreated Ti mostly through pseudopodia but partially also by lamellipodia, indicating a high degree of activation. Individual platelets and aggregates are evenly distributed throughout the Ti surface. Contrarily, mostly individual platelets in the early dendritic stage established filopodia connections on the surface of Ti + P (Figure 4c,d). It can be seen that platelet filopodia had started to extend, demonstrating a spreading tendency; however, this tendency is higher in the case of untreated Ti. Evenly distributed individual platelets can be observed on the surface of Ti HT and Ti HT + P. On the Ti HT surface spread platelets can be seen (Figure 4e,f), while on the surface of Ti HT + P platelets are round to spread the dendritic form (Figure 4g,h). However, fully spread platelets, which indicate the most active form, were not observed on these surfaces. It should be emphasized that the number of platelets on Ti HT and Ti HT + P surfaces are significantly lower compared to Ti and Ti + P, and they do not aggregate, which reduces the risk of thrombosis.

**Figure 5:**
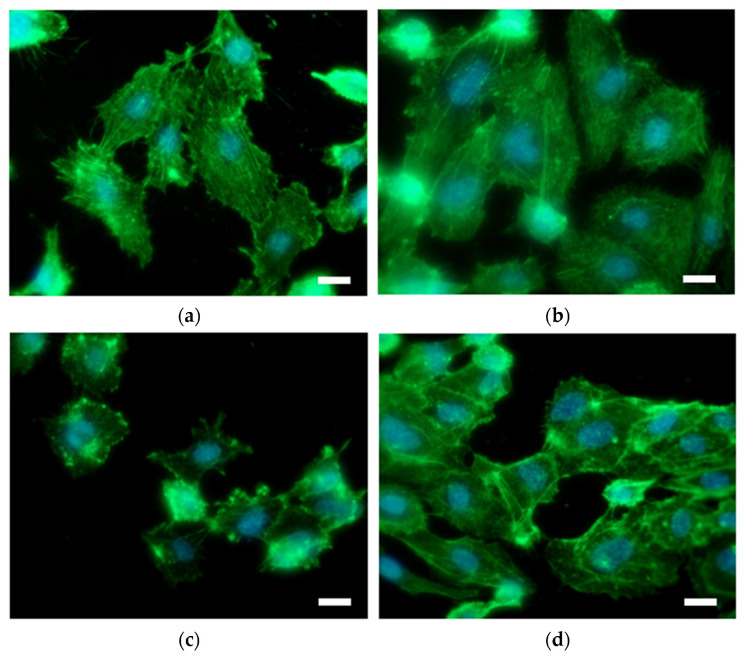
EC on the surface of (**a**) Ti foil, (**b**) plasma-treated Ti foil (Ti + P), (**c**) hydrothermally treated Ti foil (Ti HT) and (**d**) hydrothermally/plasma-treated Ti foil (Ti HT + P) determined by immunofluorescent microscopy. F-actin is shown in green (Fluorescein Phalloidin). Nuclei are visualized with DAPI (blue color). Scale bar = 25 µm.

Immunofluorescent microscopy was used to study endothelial and smooth muscle cell morphology on differently prepared titanium substrates. As good endothelialization of vascular stents is highly desired, the interaction of EC with the surface was performed. On the other hand, SMC growth, which causes restenosis, should be reduced. EC on the untreated Ti and Ti + P exhibit a physiological phenotype; however, the surface is not homogenously covered with cells in the case of pristine Ti (Figure 5a). On the plasma-treated Ti, cells are well spread and no blebbing of cells is observed (Figure 5b). On hydrothermally treated Ti foil (Ti HT), EC are irregularly distributed and clustered; some exhibit increased stress, observed by the elevated number of blebbing cells (Figure 5c), and the surface coverage is poor. However, the interaction of EC with the plasma-treated HT surface (Ti HT + P) is significantly improved as the attachment and proliferation of EC on these surfaces is enhanced, and the cells are numerous and elongated (Figure 5d).

**Figure 6:**
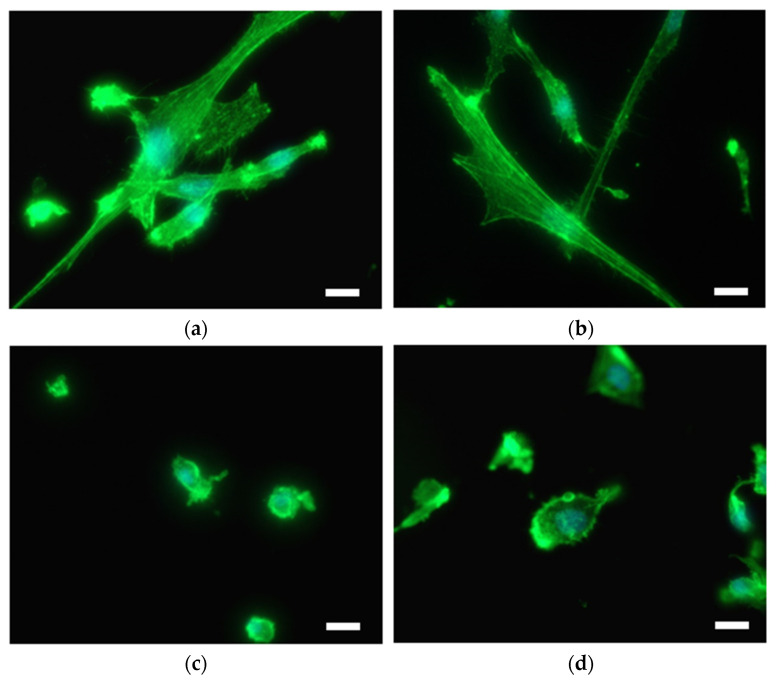
SMC on the surface of (**a**) Ti foil, (**b**) plasma-treated Ti foil (Ti + P), (**c**) hydrothermally treated Ti foil (Ti HT) and (**d**) hydrothermally/plasma-treated Ti foil (Ti HT + P), determined by immunofluorescent microscopy. F-actin is shown in green (Fluorescein Phalloidin). Nuclei are visualized with DAPI (blue color). Scale bar = 25 µm.

Extensive SMC adhesion and proliferation lead to vascular occlusion in atherosclerosis and stent restenosis. Therefore, the interaction of SMC with as-prepared materials was examined. SMC are readily attached to the surface of Ti and Ti + P; cells are elongated, mostly with non-disrupted cytoplasm (Figure 6a,b). Cells were observed over the whole Ti and Ti + P surface. On the contrary, cells were not well-attached to the surface of Ti HT and Ti HT + P (Figure 6c,d); membrane protrusions (blebbs) can be observed for all SMC on these surfaces.

## 3. Discussion

In the present paper, the biological performance of hydrothermal and plasma-treated Ti surfaces was examined. SEM and AFM analysis showed that the hydrothermal treatment enables the formation of a homogeneous nanostructured surface layer on Ti. After plasma treatment, no changes in surface morphology nor surface roughness were detected, while an increase in oxygen was observed on all plasma-treated surfaces.

The elemental composition of surfaces was obtained by XPS and EDX analysis. The main difference in elemental composition was between plasma-treated and non-plasma-treated samples, as a significant increase in oxygen and a decrease in carbon was observed, due to the oxidation of the surface and the removal of carbon contamination by reactive oxygen species.

Depth profile analysis suggests that a natively formed oxide in the depth of around 3 nm is present on Ti foil, while, on the surface of Ti HT, a thicker oxide layer exists, which is also in accordance with XPS depth analysis. According to EDX analysis, it could also be presumed that a thicker oxide layer is formed after treatment of Ti HT in plasma (Ti HT + P), as a slightly higher concentration of oxygen was detected on these surfaces (62.5 at% for plasma-treated HT and 56.5 at.% for HT). For instance, no oxygen can be observed on the surface of untreated Ti.

Since the wettability of the surface can influence the adhesion of biological cells, WCA measurements were performed. Untreated Ti is hydrophobic, while Ti + P, Ti HT and Ti HT + P are superhydrophilic.

It can be observed that numerous aggregated platelets readily attach on the surface of Ti with lamellipodia and filopodia, which is correlated with high platelet activation and a high risk of thrombosis. Individual platelets in the early dendritic form attach on the surface of Ti + P, which indicates that a surface treated by non-thermal plasma inhibits the formation of aggregates. No aggregated platelets were observed on Ti HT and Ti HT + P, and the number of attached platelets was lower. Additionally, they were mostly in the round to the spread dendritic phase, which indicates lower platelet activation on these surfaces. It is believed that such surfaces will, to a lesser extent, elicit undesired thrombus formation in comparison to the untreated Ti foil. The surface that does not promote platelet aggregation is of significant importance for medical devices, because the risk of thrombosis on such surfaces is reduced.

To evaluate the potential risk of restenosis and also proper endothelialization on the surfaces, the adhesion and proliferation of human coronary artery cells (EC and SMC) were evaluated. Adhesion and proliferation of EC on the plasma-treated samples (Ti + P and Ti HT + P) were enhanced in comparison to untreated Ti and Ti HT. Thus, plasma treatment is of significant importance for surfaces used as vascular stents, since it influences the long-term success of implantation; proper endothelialization presents an ideal antithrombogenic layer and as such allows for the better integration of the stent/implant into the human body. Contrarily, highly shrunk SMC with non-typical morphology and membrane blebbing, (possibly representing apoptotic cells) can be observed on the surface of Ti HT and Ti HT + P. These results suggest that HT surfaces inhibit the adhesion and proliferation of SMC, which would prevent uncontrolled growth of SMC after stent implantation (restenosis).

To sum up, it has been shown that the altered physicochemical properties of the Ti surface, specifically morphology, surface roughness, wettability and chemical composition, influence a material’s bio-performance. A combination of hydrothermal treatment and treatment with highly activated oxygen species (oxygen plasma treatment) resulted in the formation of a nanostructured titanium oxide surface. It has been already shown that nanotopography influences interactions with blood platelets [40,41]. For instance, Junkar et al. showed that platelets readily adhere to Ti, but the attachment and activation was not so intensive on the surface of a TiO_2_ nanotubular layer formed on the Ti substrate [19]. Authors also showed that the best-performing surfaces were nanostructured (TiO_2_ nanotubes) and were additionally treated by oxygen plasma. Higher oxygen content on the surface was found to be beneficial for the improved hemocompatibility of the materials [42]. Besides, Moradi et al. [43] showed that platelets readily adhere to the hydrophobic surface compared to the hydrophilic stainless steel and titanium surface. This was partially confirmed also in the present study, since platelet aggregates were observed on the surface of Ti (WCA = 97.6°), while much lower platelet aggregation was observed on the superhydrophilic plasma-treated titanium surface; however, other surface features, like surface nanotopography and chemistry, as mentioned before, should also be considered. Reduced platelet adhesion and aggregation observed on Ti + P, Ti HT and Ti HT + P could significantly reduce the risk of thrombosis; at the site of injury, which is common in stenting procedures, platelets quickly adhere and start to form a plug and blood coagulation that leads to vessel occlusion. These conditions can further lead to heart attack and stroke. It has been also shown that Ti HT and Ti HT + P promote the normal adhesion and growth of EC and simultaneously inhibit the adhesion and growth of smooth muscle cells. On the contrary, plain Ti substrate and Ti + P enable moderate endothelialization, but at the same time provide an appropriate environment for the proliferation of SMCs, which could present a risk of restenosis.

## 4. Materials and Methods

### 4.1. Materials

Ti foil (thickness: 0.10 mm, Advent, 99.6+%), ethanol (96% and absolute, Sigma Aldrich, St. Louis, MO, USA), Ti titanium(IV) isopropoxide, potassium hydroxide (reagent grade, 90%, flakes, Sigma Aldrich, St. Louis, MO, USA), phosphate-buffered saline PBS (tablets, Sigma Aldrich, St. Louis, MO, USA), ultra-pure deionized water (miliQ, Merck KGaA, Darmstadt, Germany), glutaraldehyde—GA (25% in H_2_O, Sigma Aldrich, St. Louis, MO, USA), fluorescein phalloidin (Molecular Probes, Thermo Fisher Scientific, Waltham, MA, USA), Triton X-100 (Sigma Aldrich, St. Louis, MO, USA), 4′,6-diamidino-2-phenylindole—DAPI (Sigma Aldrich, St. Louis, MO, USA), SlowFade reagent (Thermo Fisher Scientific, Waltham, MA, USA).

### 4.2. Hydrothermal Synthesis

Aqueous suspensions (60 mL) containing 0.5 mL of titanium (IV) isopropoxide were prepared; the potassium hydroxide (Sigma-Aldrich) was used to adjust the pH of the suspension to 10. The as-prepared suspension was transferred to a Teflon vessel containing Ti foil (10 × 10 mm) and placed in a steel autoclave (Paar, Ashland, VA, USA). The sealed autoclave was heated in an electric furnace, warmed up at a rate of 10 °C/min, maintained at 200  °C for 24 h and was then cooled to room temperature naturally. After the hydrothermal synthesis, Ti foil was washed with deionized H_2_O, dried under a stream of N_2_ and additionally dried in an oven in an air atmosphere at 70 °C for 2 h. Ti foil was then ultrasonicated for 5 min, and the washing process with deionized H_2_O, N_2_ and drying in an oven was repeated.

### 4.3. Plasma Treatment

Samples (Ti and Ti HT) were treated with oxygen plasma in the plasma reactor and evacuated with a two-stage oil rotary pump with a nominal pumping speed of 4.4 × 10^−3^ m^3^/s. Plasma was created with an inductively coupled RF generator, operating at a frequency of 13.56 MHz and an output power of about 200 W. The system parameters were measured with a double Langmuir probe and a catalytic probe [44,45]. Commercially available oxygen was leaked into the discharge chamber, a Pyrex cylinder with a length of 0.6 m and an inner diameter of 0.036 m. The pressure was measured by an absolute vacuum gauge, and it was adjusted during continuous pumping by a precise leak valve. The pressure in our experiments was fixed at 75 Pa, since at this value the highest degree of dissociation of gaseous molecules measured by the catalytic probes was detected. Plasma with an ion density of about 2 × 10^15^ m^−3^, thermal energy of 4 eV and a neutral atoms density of about 4 × 10^21^ m^−3^ was obtained at these discharge parameters. The samples placed on the quartz glass holder were treated for 60 s.

### 4.4. Characterization

#### 4.4.1. Scanning Electron Microscope (SEM) Analysis

The morphological and compositional analysis of the materials was conducted by scanning electron microscopy (JEOL JSM-7600F) and energy-dispersive X-ray spectroscopy (Oxford Instruments, Abingdon-on-Thames, UK). For the biological evaluation of platelets on the surface, the samples were coated with gold/palladium and examined by SEM at an accelerating voltage of 5 kV. The test was done in triplicate, and only representative images are shown.

#### 4.4.2. Atomic Force Microscopy Analysis (AFM)

Topographic features of the samples were examined by atomic force microscopy (Solver PRO, NT MDT) in tapping mode in air. Samples were scanned with the standard Si cantilever with a force constant of 22 N/m and at a resonance frequency of 325 kHz (the tip radius was 10 nm, and the tip length was 95 μm) and the scan rate set to 1.3 Hz.

#### 4.4.3. X-ray Photoelectron Spectroscopy (XPS)

The X-ray photoelectron spectroscopy (XPS) analyses were carried out on the PHI-TFA XPS spectrometer produced by Physical Electronics Inc. (Chanhassen, MN, USA). Samples were put on the sample holder and were introduced into the ultra-high-vacuum spectrometer. The analyzed area was 0.4 mm in diameter, and the analyzed depth was about 3–5 nm. This high surface sensitivity is a general characteristic of the XPS method. Sample surfaces were excited by X-ray radiation from a monochromatic Al source at a photon energy of 1486.6 eV. The high-energy resolution spectra were acquired with an energy analyzer operating at a resolution of about 0.6 eV and pass energy of 29 eV. During data processing, the spectra from the surface were aligned by setting the C 1s peak at 285.0 eV, characteristic for C-C bonds. The accuracy of binding energies was about ±0.3 eV. The quantification of surface composition was performed from XPS peak intensities taking into account relative sensitivity factors provided by the instrument manufacturer. Three different XPS measurements were performed on each sample, and the average composition was calculated.

The XPS method was utilized to obtain information about the chemical composition of Ti and Ti HT in-depth. To determine chemical composition in-depth, an Ar+ ion beam with 1 keV energy was used for sputtering at an incidence angle of 45° and a raster of 5 mm × 5 mm. The sputtering rate was approximately 1 nm/min.

#### 4.4.4. Water Contact Angle (WCA) Measurements

The surface wettability was performed with a Drop Shape Analyser DSA-100 (Krüss GmbH, Hannover, Germany) by a sessile drop method to measure a static contact angle. The contact angle on the surface was analyzed immediately after plasma treatment by adding a 2.5 µL drop of deionized water on 8 different areas of the surface. Three measurements were performed for each sample, and the average value was calculated. The relative humidity was around 45%, and the operating temperature was 21 °C, which did not vary significantly during continuous measurements.

### 4.5. Incubation of the Samples with Whole Blood

The adhesion and activation of platelets on the samples was done according to the following procedure. Tests were performed by following the Declaration of Helsinki and approved by Slovenia’s Ethics Committee (number of approval 56/03/10). Prior to whole blood incubation, samples were cleaned with ethanol, dried and incubated with whole blood taken by vein puncture from a healthy human donor. The blood was drawn into 9 mL tubes coated with tri-sodium citrate anticoagulant. Afterward, the fresh blood (250 μL) was incubated with samples in 24-well plates for 45 min at room temperature. After incubation, 250 μL of phosphate-buffered saline (PBS) was added to the whole blood. The blood with PBS was then removed, and the titanium surface was rinsed 5 times with 250 μL PBS in order to remove weakly adherent platelets. Adherent cells were subsequently fixed with 250 μL of 0.5% GA (glutaraldehyde) solution for 15 min at room temperature. Afterward, the surfaces were rinsed with PBS and then dehydrated using a graded ethanol series (50, 70, 80, 90, 100 and again 100 vol. % ethanol) for 5 min and in the last stage in the series (100 vol. % ethanol) for 15 min. Then the samples were placed in a critical point dryer, where the solvent is exchanged with liquid carbon dioxide. By increasing the temperature in the drier, the liquid carbon dioxide passes the critical point, at which the density of the liquid equals the density of the vapor phase. This drying process preserves the natural structure of the sample and avoids surface tension, which could be caused by normal drying. The dried samples were subsequently coated with gold/palladium and examined by means of SEM. The test was done in triplicate, and only representative images are shown in this paper.

### 4.6. Cell Culture

Human coronary artery endothelial cells (EC) were purchased from Lifeline Cell Technology (Frederick, MD, USA) and human coronary artery smooth muscle cells (SMC) were purchased from ProVitro AG (Berlin, Germany). EC and SMC were plated into 75 cm^2^ flasks (TPP, Trasadigen, Switzerland) at 37 °C in a humidified atmosphere at 5% CO_2_ and grown in VascuLife EnGS endothelial medium complete kit (Frederick, MD, USA) and smooth muscle cell growth medium FCS-kit (ProVitro AG, Berlin, Germany) respectively, following the manufacturer’s instructions. For experiments, subconfluent cell cultures were used between passages 4 and 6.

### 4.7. Immunofluorescent Microscopy and Cell Morphology

The EC and SMC were seeded on sample materials in 12-well plates at a density of −20 × 10^3^ cells per cm^2^ and grown for 2 days. The test was conducted in biological triplicate. Staining with Fluorescein Phalloidin (Molecular Probes, Thermo Fisher Scientific, Waltham, MA, USA) was performed following the manufacturer’s instructions. Briefly, cells were washed 2 times for 3 min with PBS at pH 7.4, fixed in 3.7% formaldehyde solution for 10 min and washed 3× for 3 min with PBS at room temperature. Cells were incubated in detergent 0.1% Triton X-100 for 4 min then washed with PBS 3 times for 3 min. Dye stock was diluted 1:40 in PBS with 1% BSA and applied to EC and SMC for 30 min. The final washing steps were performed 3 times for 3 min with PBS. DAPI (4′,6-diamidino-2-phenylindole)-staining (Molecular Probes, Thermo Fisher Scientific, Waltham, MA, USA) was performed following manufacturer’s instructions. Briefly, samples were incubated with 300 nM DAPI in PBS for 5 min and washed with PBS, for 3 min at room temperature. SlowFade reagent (Thermo Fisher Scientific, Waltham, MA, USA) was applied to EC and SMC (1 drop), and a cover slip was fixed on top with clear nail polish. Slides were examined and/or stored in the dark at 4 °C. Images were generated using the fluorescent microscope Nikon Eclipse E400 and a digital camera (Nikon Instruments, Dusseldorf, Germany). Analysis was performed with Nikon ACT-1 imaging software, and the representative images are presented.

## 5. Patents

Patent application resulting from the work reported in this manuscript: Junkar, I., Benčina, M., Zaplotnik, R., Mozetič, M., Sodin-Šemrl, S., Lakota, K., Iglič, A. Method for treatment of medical metals and their alloys, EU 21 159 240.7. München: European Patent Office, 25 February 2020.

## Figures and Tables

**Table 1:** Surfaces prepared by different treatment methods, abbreviations of the samples with corresponding data on WCA.

Surface	Abbreviatio	WCA (°)
Titanium	Ti	97.6
Plasma treated titanium	Ti + P	<5
Hydrothermally treated titanium	Ti + HT	<5
Hydrothermally and plasma treated titanium	Ti + HT + P	<5

**Table 2:** Chemical composition of the surface of materials examined by XPS.

	(Atomic %)			Ratio (XPS)	
Material	Ti	O	C	Ti/O	C/O
Ti	19.5	48.5	32.0	0.40	0.61
Ti + P	16.0	68.5	15.5	0.23	1.03
Ti HT	20.0	48.7	31.3	0.41	0.64
Ti HT + P	22.3	61.1	16.6	0.36	0.27

## Data Availability

All important data is included in the manuscript.
